# Comparison of White Light With Narrow Band Imaging Using Flexible Laryngoscopy for the Detection of Local Recurrences After (Chemo)Radiation for Pharyngeal or Laryngeal Cancer: A Randomised Controlled Trial

**DOI:** 10.1111/coa.14293

**Published:** 2025-02-14

**Authors:** Constanze Scholman, Jeroen M. Westra, Manon A. Zwakenberg, Jan Wedman, Bert van der Vegt, Roel J. H. M. Steenbakkers, Sjoukje F. Oosting, Gyorgy B. Halmos, Bernard F. A. M. van der Laan, Boudewijn E. C. Plaat

**Affiliations:** ^1^ Department of Otorhinolaryngology – Head & Neck Surgery University of Groningen, University of Medical Center Groningen Groningen the Netherlands; ^2^ Department of Pathology and Medical Biology University of Groningen, University of Medical Center Groningen Groningen the Netherlands; ^3^ Department of Radiation Oncology University of Groningen, University of Medical Center Groningen Groningen the Netherlands; ^4^ Department of Medical Oncology University of Groningen, University of Medical Center Groningen Groningen the Netherlands; ^5^ Department of Otorhinolaryngology – Head & Neck Surgery Haaglanden Medical Center The Hague the Netherlands

**Keywords:** clinical outcome, flexible laryngoscopy, head and neck cancer, narrow band imaging, randomised controlled trial, recurrence

## Abstract

**Objectives:**

Early detection of local recurrences in patients with head and neck squamous cell carcinoma (HNSCC) is crucial for long‐term survival. Aim of this study was to compare white light (WL) imaging with narrow band imaging (NBI) during flexible laryngoscopy for detection of local recurrences and evaluate the effects on clinical outcome in patients with HNSCC treated with radiotherapy ± chemotherapy ((C)RT).

**Design:**

Prospective randomised controlled trial.

**Setting:**

Tertiary head and neck oncologic center.

**Participants:**

257 patients without residual disease after (C)RT were randomised in a WL group (*n* = 120) or WL‐NBI group (*n* = 137) and followed for 24 months.

**Main Outcome Measures:**

Local recurrence rate and overall survival, disease‐specific survival, disease‐free survival and local recurrence‐free survival were compared between both groups.

**Results:**

Detection rate of local recurrences was the same in both groups: 11.7% in WL‐NBI and 10.0% in WL (*p* > 0.05). Overall survival (WL: 88.3%, WL‐NBI: 87.6%), disease‐specific survival (WL: 86.7%, NBI: 83.9%), disease‐free survival (WL: 85.0%, WL‐NBI: 83.2%) and local recurrence‐free survival (WL: 90.0%, WL‐NBI: 89.1%) showed no superiority in the WL‐NBI group (*p* > 0.05).

**Conclusions:**

Local recurrence rates are relatively low in the first 24 months after (C)RT for HNSCC. WL‐NBI did not improve the detection of local recurrences nor the survival compared to WL.

**Trial Registration:** This randomised clinical trial was registered at the research register UMCG with the number 201500918

**Summary**

- A randomised controlled trial in a tertiary head and neck oncologic center comparing white light (WL) imaging with narrow band imaging (WL‐NBI) during flexible laryngoscopy for detection of local recurrences and evaluating effects on clinical outcome in patients with pharyngeal or laryngeal carcinoma treated with radiotherapy ± chemotherapy ((C)RT).
- 257 patients without residual disease after (C)RT were randomised in a WL group (*n* = 120) or WL‐NBI group (*n* = 137) and followed for 24 months.
- Detection rate of local recurrences was the same in both groups: 11.7% in WL‐NBI and 10.0% in WL (*p* > 0.05).
- Overall survival, disease‐specific survival, disease‐free survival and local recurrence‐free survival showed no superiority in the WL‐NBI group.
- WL‐NBI did not improve the detection of local recurrences nor the survival compared to WL.

## 1 Introduction

Early detection of local recurrences or second primary tumours (SPT) in patients with head and neck squamous cell carcinoma (HNSCC) is crucial for long‐term survival [1]. The recurrence rate of HNSCC depends on tumour stage and treatment modality of the patient [2, 3, 4, 5, 6, 7]. In the Netherlands, recurrence rate within 5 years varies from 4% to 12% for local recurrences of oropharyngeal, hypopharyngeal or laryngeal cancer [2]. However, data on specific local recurrence rates after radiotherapy (RT) are scarce. Large meta‐analyses present the mostly combined rate of local and regional recurrence [8]. As a result of long‐term mutagenic insults, multiple precancerous lesions can develop in the field surrounding the primary tumour and possibly progress into SPT [9].

For endoscopy, conventional white light (WL) laryngoscopy can be combined with narrow band imaging (NBI) [10]. NBI has become a frequently used tool to visualise an enhanced image of neoangiogenic blood vessel patterns in the upper aerodigestive tract [10]. Also, the NBI detected abnormal blood vessel patterns showing intrapapillary capillary loops (IPCL), which are associated with malignant mucosal changes [11]. Several studies showed the beneficial effects of using NBI in the detection of primary HNSCC and upper aerodigestive tract lesions by increasing diagnostic accuracy and inter‐ and intraobserver reliability compared to WL [12, 13, 14]. However, the detection of local recurrences or SPTs can be difficult since radiotherapy causes chronic inflammation, oedema and hypervascularisation of the irradiated mucosa [11]. Capillaries are damaged, resulting in disarrangement of normal blood vessels. Therefore, using NBI to differentiate between pathological IPCLs and disturbed blood vessel patterns after (chemo)radiation is challenging. Cosway et al. reported a high sensitivity of 95%–98% and specificity of 96%–99% for detecting a recurrence of HNSCC after radiotherapy with NBI [15]. However, additional value of adding NBI to WL in patients with pharyngeal or laryngeal cancer on local recurrence detection compared to WL alone are unknown [16]. Early detection of local recurrences could influence survival rates [17, 18]. The aim of this randomised clinical study was to evaluate the additional value of NBI during follow‐up of patients treated for pharyngeal and laryngeal cancer with radiotherapy with or without concomitant chemotherapy ((C)RT) on the detection rate of local recurrences and on clinical outcome.

## 2 Methods and Materials

### 2.1 Ethical Considerations

The Institutional Review Board/METC of the University of Medical Center Groningen approved this study (research register 201 500 918), according to the Dutch Act on Medical Research involving Human Subjects. The CONSORT guideline was followed for this study.

After informed consent, patients were enrolled between November 2014 and 2021 at the University Medical Center Groningen. Clinical outcome data were last verified in January 2023. Patients with a proven squamous cell carcinoma of the oropharynx, hypopharynx or larynx were included at the Department of Otolaryngology and Head and Neck Surgery during the first visit after curative intended (C)RT treatment. Patients treated with cetuximab or additional immunotherapy were excluded.

Disease was staged according to the TNM 7 classification. RT consisted of simultaneous integrated boost (SIB) using intensity‐modulated radiotherapy (IMRT), volumetric modulated arc therapy (VMAT) or intensity‐modulated protontherapy (IMPT) with a total dosage of 70Gy. Concomitant chemotherapy consisted of weekly cisplatin 40 mg/m^2^ or carboplatin 300–350 mg/m^2^ at Day 1, 22 and 43 followed by 5‐fluorouracil 600 mg/m^2^/day for 96 h. Local and regional response status were evaluated by CT or MRI 6–8 weeks after completing treatment, followed by ^18^F‐FDG‐PET in case of doubt at 12 weeks posttreatment. Patients were excluded in case residual disease was revealed by response imaging. Local response status of early glottic cancer (T1 stage) was determined by clinical follow‐up.

### 2.2 Follow‐Up of the Patients

All patients underwent at least two flexible laryngoscopic examinations during follow‐up. The minimal length of follow‐up was 24 months. According to local standard of care, follow‐up alternated every 3 months between the Otolaryngology and the RT department. NBI was only performed at follow‐up visits to the Otolaryngology and Head and Neck Surgery department. Flexible video‐endoscopy was performed using a rhinolaryngoscope ENF VH (Olympus Medical Systems, Tokyo, Japan) attached to a HD monitor (XION, Modelcode MD‐2409LP, serial number 121624012, build in 2012, XION medical GmbH, Berlin, Germany). All endoscopy images and videos were recorded and stored in the electronic patient records. Patients were randomised into two groups using Microsoft Excel: WL group, receiving only WL examination using flexible laryngoscopy and NBI group, undergoing WL with NBI during flexible laryngoscopy at 3, 9, 15, 21 and 27 months after treatment. Diagnostic procedures were not blinded.

The pharynx and larynx were examined according to general practice. In the NBI group all anatomic subsites were each first visualised with WL and subsequently with NBI through near contact imaging. All laryngoscopy images were assessed by ENT specialists, experienced in WL and NBI flexible laryngoscopy. WL was regarded as suspicious of malignancy based on the physician's expertise. Lesions were examined accounting for, i.e. erythema, irregularities and mucosa elevation. NBI was regarded as suspicious for malignancy in case of blood vessel patterns IPCLs according to Ni et al. classification type V lesions [19]. Upon malignancy suspicion, biopsies were taken under general anaesthesia with direct laryngoscopy or under local anaesthesia with flexible laryngoscopy for histopathological confirmation.

Patient, tumour, treatment and follow‐up characteristics were collected from the electronic patient files and stored pseudonymised. Primary endpoint was detection of local recurrences defined as a newly developed tumour in the irradiated area of the primary treated tumour. Secondary endpoint was oncologic outcome assessed by overall survival, disease‐specific survival, disease‐free survival and local recurrence‐free survival.

### 2.3 Statistical Analysis

Descriptive statistics of patient demographics and clinical characteristics were reported as frequencies (proportions) for categorical variables and median (range min–max) for continuous variables. The Pearson chi‐square test or Fisher's exact test were used to analyse baseline patient characteristics. The Kaplan–Meier analysis was used to calculate survival rates and survival curves and the log‐rank test compared survival between the two study groups. SPSS version 28 statistical software (IBM Corp., Armonk, NY) was used to perform statistical analyses. A *p*‐value < 0.05 was considered statistically significant.

## 3 Results

### 3.1 Patient Demographics and Clinical Characteristics

**FIGURE 1:**
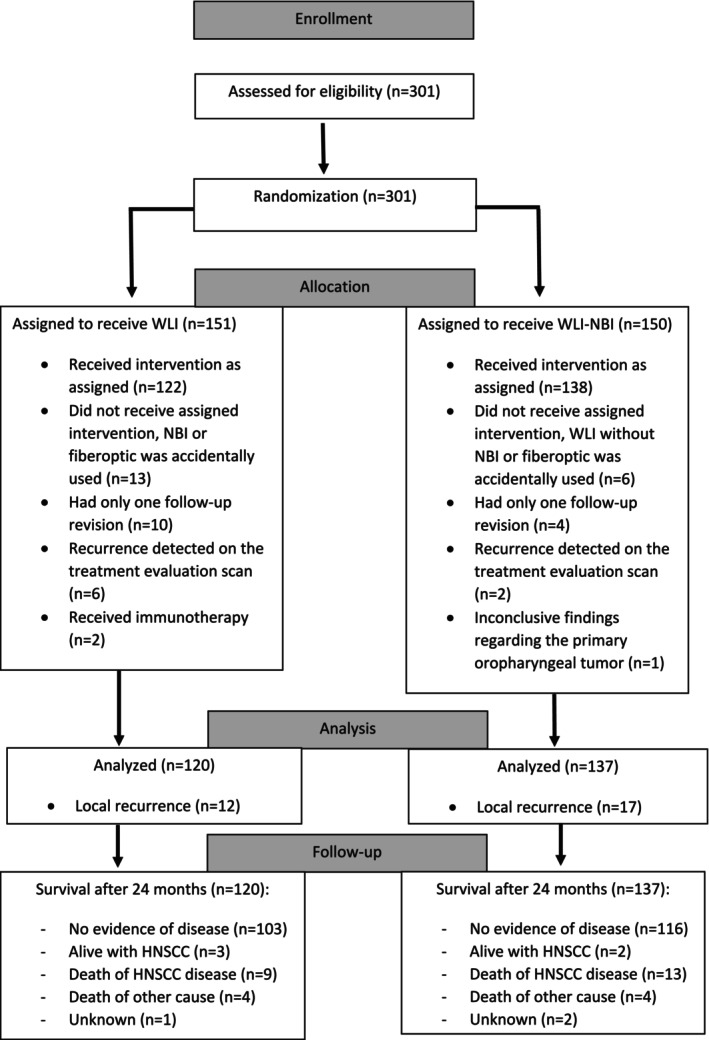
Flow diagram of study population.

**FIGURE 2:**
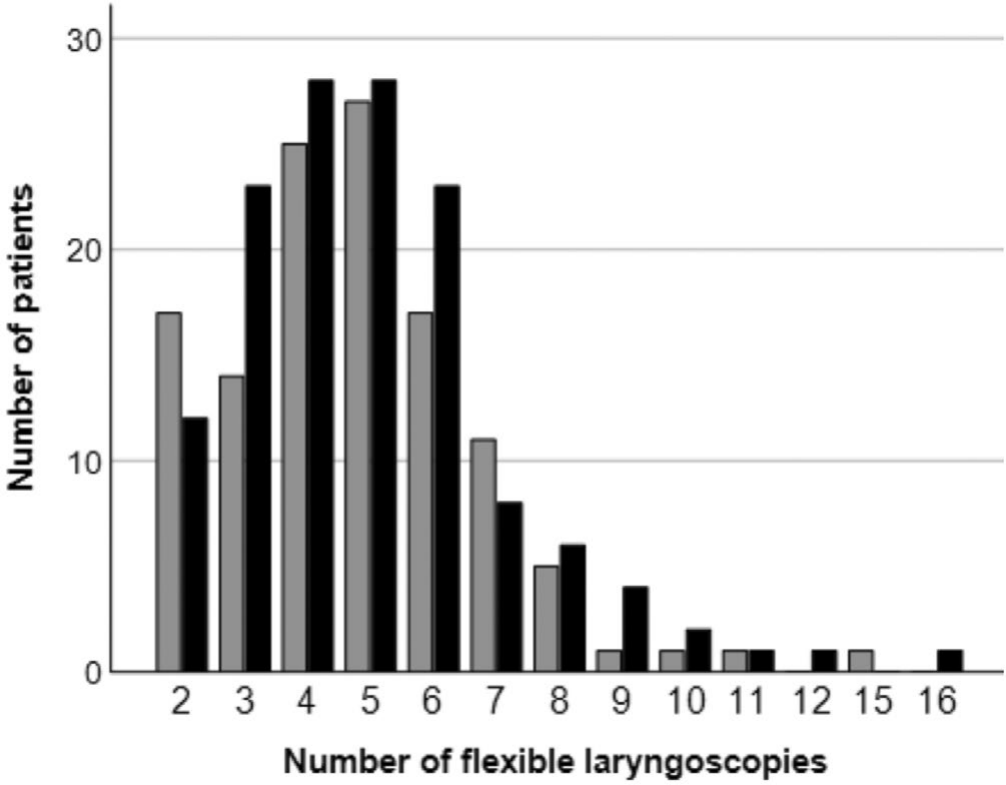
Mean number of flexible laryngoscopies for white light imaging group (grey) and white light—narrow band imaging group (black).

Overall, 301 patients were assessed for enrolment (see flow diagram in Figure 1). In total, 44 patients were excluded: during follow‐up visits, 19 patients were not examined consistently with the imaging modality for which the patient was randomised, 14 patients had only one revision, 8 patients showed a recurrence on the evaluation scan and 2 patients received cetuximab. One patient was excluded based on radiological imaging for a regional recurrence of an oesophageal tumour with inconclusive findings regarding the primary oropharyngeal tumour treated for. Demographics and clinical characteristics of the remaining 257 patients are demonstrated in Table 1: 120 patients underwent WL and 137 patients received WL with NBI during follow‐up (see Figure 2 for mean number of flexible laryngoscopies per group). The majority of included patients were men (79.8%). Most tumours were located in the larynx (61.1%) and oropharynx (29.2%). The most common tumour stages were stage IVa (40.1%) and stage III (26.8%), accounting for more than two‐thirds of the study group. Most patients were treated with IMRT (39.7%). Almost one‐third of the patients received (C)RT (31.9%). Around 45% of patients had lymph node metastasis.

**TABLE 1:** Patient demographics and clinical characteristics.

Patients characteristics	Total group	WL group	WL‐NBI group	*p*
Gender				0.82
Male	205 (79.8%)	95 (79.2%)	110 (80.3%)	
Female	52 (20.2%)	25 (20.8%)	27 (19.7%)	
Median age in years ± SD [range]	65 **±** SD 9.5, [32.3–90.2]	66 **±** SD 9.1, [32.3–86.0]	64.4 **±** SD 9.8, [40.9–90.2]	0.49
Location tumour				5.68 (0.13)
Larynx	157 (61.1%)	76 (63.3%)	81 (59.1%)	
Oropharynx	75 (29.2%)	30 (25%)	45 (32.8%)	
Hypopharynx	25 (9.7%)	14 (11.7%)	11 (8.0%)	
TNM 7 stage				7.29 (0.20)
I	41 (15.9%)	14 (11.7%)	27 (19.9%)	
II	36 (14.0%)	15 (12.5%)	21 (15.3%)	
III	69 (26.8%)	35 (29.2%)	34 (24.8%)	
IVa	103 (40.1%)	53 (44.2%)	50 (36.5%)	
IVb	8 (3.1%)	3 (2.5%)	5 (3.6%)	
Tumour stage				6.53 (0.9)
Tcis/T1	57 (22.2%)	20 (16.7%)	37 (27.0%)	
T2	56 (21.8%)	23 (19.2%)	33 (24.1%)	
T3	95 (37.0%)	50 (41.7%)	45 (32.8%)	
T4	49 (19.1%)	27 (22.5%)	22 (16.1%)	
Lymph node metastasis				1.97 (0.85)
N+	115 (44.7%)	58 (48.3%)	57 (41.6%)	
N0	142 (55.3%)	62 (51.7)	80 (58.4%)	
Radiotherapy treatment modality				0.73 (0.95)
IMRT‐SIB	102 (39.7%)	47 (39.2%)	55 (40.1%)	
VMAT‐SIB	59 (23.0%)	26 (21.7%)	33 (24.1%)	
VMAT	56 (21.8%)	26 (21.7%)	30 (21.9%)	
IMPT	36 (14.0%)	19 (15.8%)	17 (12.4%)	
IMRT	4 (1.6%)	2 (1.7%)	2 (1.5%)	
Concurrent chemotherapy				2.4 (0.13)
Yes	82 (31.9%)	44 (36.7%)	38 (27.7%)	
No	175 (68.1%)	76 (63.3%)	99 (72.3%)	
Total group	257 (100%)	120 (46.7%)	137 (53.3%)	

Abbrevations: IMPT, intensity‐modulated protontherapy; IMRT, intensity‐modulated radiotherapy; NBI, narrow band imaging; SIB, simultaneous integrated boost; VMAT, volumetric modulated arc therapy; WL, white light imaging.

* *p* value comparing WL group to WL‐NBI group.

### 3.2 Detection of Local Recurrences With WL and WL‐NBI

In both groups, mean number of flexible laryngoscopies was 5, ranging from 2 to 15 laryngoscopies with WL, and from 2 to 16 laryngoscopies with WL‐NBI. In the WL group, 31 patients and in the WL‐NBI group 32 patients were suspected of a local recurrence during laryngoscopic examination and biopsies were taken. As shown in Table 2, 12 local recurrences were confirmed in the WL group, indicating a positive predictive value of 39.7%. In the WL‐NBI group, 16 local recurrences were confirmed, demonstrating a positive predictive value of 50%. Local recurrence rate was not significantly different between WL and WL‐NBI group (*p* > 0.05).

**TABLE 2:** Detection of recurrences in both white light imaging group (*n* = 120) and white light—narrow band imaging group (*n* = 137).

	Total group	WL group	WL‐NBI group	*p* (Significance)
Number of LR	28 (10.9%)	12 (10.0%)	16 (11.7)	0.67
Mean time to LR ± SD [range], months	10.1 **±** 7.8 [2.9–45.2]	8.8 **±** 5.4 [3.0–21.3]	11.2 **±** 9.6 [2.9–45.2]	0.37
Location of LR				0.85
Glottis	18 (7.0%)	7 (58.3%)	11 (64.7%)	
Oropharynx	7 (2.7%)	3 (25.0%)	4 (23.5%)	
Hypopharynx	4 (1.6%)	2 (16.7%)	2 (11.8%)	
Regional recurrence	17 (6.6%)	8 (6.7%)	9 (6.6%)	0.98
Distant metastasis	5 (2.0%)	2 (1.7%)	3 (2.2%)	0.76
Mean number of endoscopies ± SD [range]	5 **±** 2.1 [2–16]	5 **±** 2.1 [2–15]	5 **±** 2.2 [2–16]	0.72
Number of direct laryngoscopies				0.22
0	189 (73.5%)	85 (70.8%)	104 (75.9%)	
1	54 (21.0%)	28 (23.3%)	26 (19.0%)	
2	8 (6.1%)	2 (1.7%)	6 (4.4%)	
3	1 (0.4%)	1 (0.8%)	0	
FLB	5 (2.0%)	4 (3.3%)	1 (0.7%)	

Abbreviations: FLB, flexible laryngoscopic biopsy; LR, local recurrence; NBI, narrow band imaging; WL, white light imaging.

### 3.3 Clinical Outcome of Patients Using WL or WL‐NBI During Follow‐Up

**FIGURE 3:**
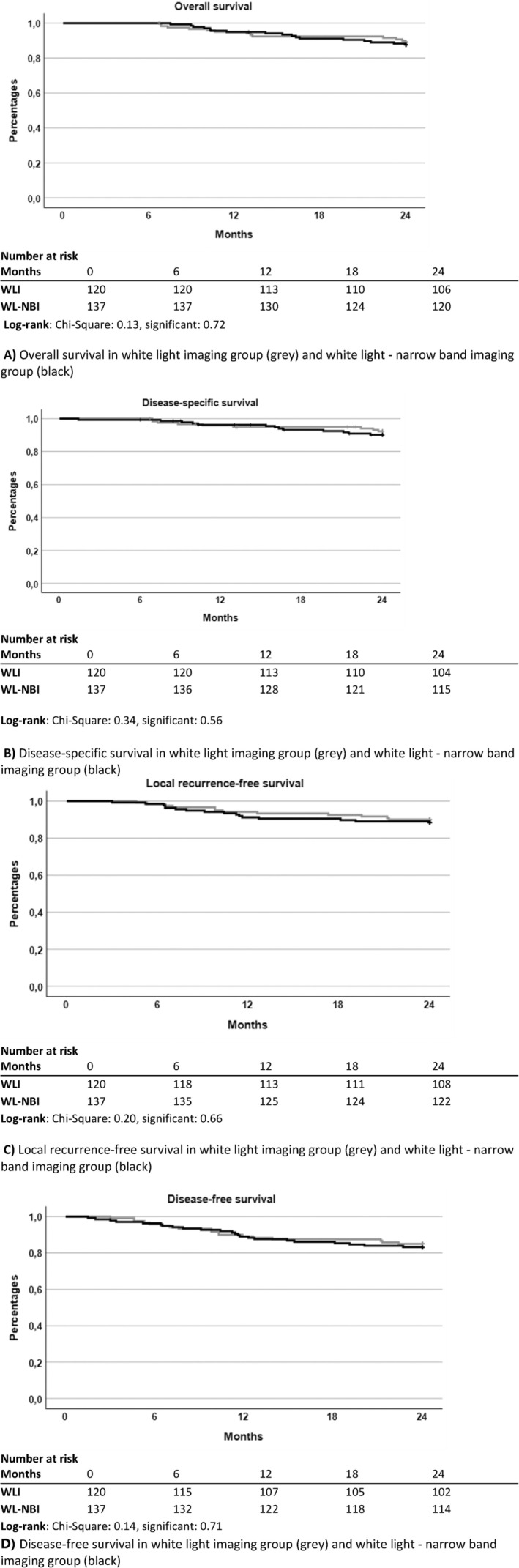
Graphs of overall survival (A), disease‐specific survival (B), local recurrence‐free survival (C) and disease‐free survival (D) in white light imaging group (grey) and white light—narrow band imaging group (black).

Overall survival after 24 months of follow‐up did not differ between patients examined with WL or WL‐NBI (88.3% in WL group vs. 87.6% in WL‐NBI group, *p* = 0.72, Figure 3A). In the WL group, 7.5% (9 patients) died because of HNSCC, compared to 9.5% (13 patients) in the WL‐NBI group, resulting in no difference in disease‐specific survival (*p* = 0.56, Figure 3B). Disease‐free survival and local recurrence‐free survival did not significantly differ between the WL and the WL‐NBI group (*p* = 0.71 and *p* = 0.66, Figure 3C,D, respectively).

## 4 Discussion

To the best of our knowledge, this is the first randomised controlled clinical trial comparing WL with WL‐NBI for the detection of local recurrences during standard oncological follow‐up of HNSCC patients initially treated successfully with (C)RT. In patients with a favourable response to (C)RT, i.e. without signs of residual disease, and who went into standard oncological follow‐up, the local recurrence rate in the first 24 months was relatively low (10.0% in WL and 11.7% in WL‐NBI). The use of WL‐NBI after RT showed no additional value compared to WL for the detection of local recurrences in patients after (C)RT. There was no difference in overall survival, disease‐specific survival or local recurrence‐free survival between both groups.

Several studies showed the beneficial effects for NBI in detecting malignant upper aerodigestive tract lesions by increasing diagnostic accuracy and inter‐ and intraobserver reliability [12, 13, 14, 15, 16, 20, 21, 22]. Cosway et al. reported a high sensitivity of 95%–98% and specificity of 96%–99% for detecting a recurrence of HNSCC after radiotherapy with NBI based on the study of Lin et al. and Chu et al. [15, 21, 22]. Piazza et al. showed a higher sensitivity of using additional NBI examination than using WL alone during examination (NBI: 100% and WL: 66%) [20]. Also, Boscolo Nata et al. reported a sensitivity of 100% and specificity of 98.6% with NBI for detecting local HNSCC recurrences after RT with or without chemotherapy [16]. In these studies, no randomisation was used and all patients were examined with both WL and NBI by the same observer, often an experienced physician [16, 20, 21]. The results could be influenced by intraobserver bias [13]. Additionally, in several studies, tumour stage and treatment specifics of patients were not described [16, 20]. In the study of Boscolo Nata et al. only half of the patients received radiotherapy, while the other half received surgery. The study of Chu et al. used WL and NBI alternately, so WL was not compared to NBI. Most studies included mainly patients with tumours located in the oral cavity or oropharynx [16, 21, 22]. Therefore, these results cannot be easily translated into a standard clinical setting. Our study design of a randomised clinical trial made our study more reliable and allows us to translate our results into standard clinical settings.

NBI has been emphasised as beneficial during follow‐up because of its minimal invasive technique, examination speed and safety of the patient [16, 20]. The study of Piazza et al. endorsed NBI for being more cost effective than PET‐CT during follow‐up but did not analyse the effects of NBI for the patient's survival. However, PET‐CT or MRI should be considered complementary because of its capability of visualising deep anatomical structures and surrounding tissue. By focusing on the effects on patients’ survival and comparing two randomised groups, we acquired a complete understanding of the potential effects of using NBI.

Despite the reported high sensitivities and specificities of NBI, studies show that identification of a recurrence remains difficult due to tissue disarrangement after RT [11]. Additionally, the Ni et al. classification was suggested for changes of IPCLs due to primary tumours and not for irradiated mucosa [19, 23]. Surprisingly, the local recurrence rate of the included patient group was low (10.0% in WL and 11.7% in WL‐NBI).

The post‐treatment evaluation PET/CT scan clearly increased early detection of residual disease and therefore diminishes the detection of malignancies by laryngoscopy, underlining the value of radiological and PET response evaluation. Patients with locally stable disease or a residual disease on the treatment evaluation scan were excluded, which also lowers the recurrence rate in our study. The question arises whether systematic follow‐up using laryngoscopy is necessary to detect local recurrences in only 10% of the patients, assuming that those patients have no symptoms of a local recurrence.

We did not find differences in detecting local recurrences for patients followed with WL compared to WL‐NBI, nor in the time of diagnosis. An explanation could be the amount of time in between two subsequent examinations. The time interval of 6 months between two ENT visits may arguably be too long to notify a distinction in time of detection between NBI and WL, but that was the same in both groups due to randomisation. Despite this, a strong feature of this study was the inclusion of a homogenous group of patients without signs of local residual disease after (C)RT, which made both groups very comparable. Furthermore, the same number of direct laryngoscopies in the WL as in the WL‐NBI group (one direct laryngoscopy: 28.7% in WL and 23.9% in WL‐NBI, *p* > 0.05) showed that additional NBI did not lead to more direct laryngoscopies because of its better visualisation of abnormal blood vessels induced by radiotherapy or as a sign of local recurrence.

We did not find a difference in overall survival, disease‐free survival or local recurrence free‐survival in both groups. The survival rates of our group after 2 years of follow‐up were similar to the study of Leemans et al. (80%–90%) and slightly higher than the study of Bollen et al. (73.6%) [2, 3]. The higher overall survival of our study could possibly be explained by the well selected patient group, who had to show no signs of residual disease after (C)RT.

The strength of this study is the large prospective homogenous cohort treated in a tertiary center proficient in the use of NBI. All patients were treated with RT, resulting in minimising confounders of developing recurrences due to different treatment options.

This study has some limitations. Besides the differences in follow‐up between RT and ENT department, the COVID‐19 pandemic during this study could have influenced follow‐up visit frequency. However, this was the case in both groups, and all patients were required to have received at least two flexible laryngoscopic examinations during the follow‐up to be included. A larger multicenter study with more frequent use of NBI is needed for definitive conclusions regarding a possible benefit of additional NBI during flexible laryngoscopy in the follow‐up of irradiated HNSCC patients.

## 5 Conclusion

The local recurrence rate in HNSCC patients with no signs of residual disease after (C)RT is low in the first 24 months of follow‐up. Flexible laryngoscopy using WL‐NBI after (C)RT showed no additional value in detecting local recurrences and did not alter clinical outcome compared to WL. There is no evidence yet that NBI during follow‐up after (C)RT has a significant impact on clinical outcome.

## Author Contributions

C.S. study design; data acquisition; data analysis; interpretation; manuscript editing. J.M.W. study design; data acquisition; data analysis; interpretation; manuscript editing. M.A.Z. study design; data acquisition; data analysis; interpretation; manuscript editing. J.W. interpretation; manuscript editing. B.v.d.V. interpretation; manuscript editing. R.J.H.M.S. interpretation; manuscript editing. S.F.O. interpretation; manuscript editing. G.B.H. interpretation; manuscript editing. B.F.A.M.v.d.L. Interpretation; manuscript editing. B.E.C.P. study design; data acquisition; data analysis; interpretation; manuscript editing.

## Ethics Statement

The Institutional Review Board/METC of the University Medical Center Groningen assessed this study design and approved the protocol (research register 201 500 918), based on the Dutch Medical Research Law (Wet medisch‐wetenschappelijk onderzoek met mensen [WMO]).

## Consent

All included patients in this study have given written informed consent.

## Conflicts of Interest

Boudewijn EC Plaat had a consultancy role for and has received unrestricted research funding by Olympus Medical Systems EU. Sjoukje F. Oosting received research grants from Novartis, Pfizer, Celldex Therapeutics and Merck, paid to the institution. Advisory board for Bristol Myers Squibb and Genmab, paid to the institution. Speaker's bureau for Merck and Travel Congress Management B.V., paid to the institution. Member of steering and safety monitoring committee for ALX Oncology, unpaid. Bert van der Vegt—honoraria received by University Medical Center Groningen for expertise or scientific advisory board/consultancy (on request): Visiopharm, Philips, MSD/Merck; speaker's fee from Visiopharm, Diaceutics, and MSD/Merck.

### Peer Review

The peer review history for this article is available at https://www.webofscience.com/api/gateway/wos/peer‐review/10.1111/coa.14293.

## Data Availability

The data that support the findings of this study are available on request from the corresponding author. The data are not publicly available due to privacy or ethical restrictions.
